# Cross-Talks between Natural Killer Cells and Distinct Subsets of Dendritic Cells

**DOI:** 10.3389/fimmu.2014.00159

**Published:** 2014-04-10

**Authors:** Guido Ferlazzo, Barbara Morandi

**Affiliations:** ^1^Department of Human Pathology, University of Messina, Messina, Italy; ^2^Cellular Therapy Program, University Hospital Policlinico G. Martino, Messina, Italy; ^3^Department of Experimental Medicine, University of Genoa, Genoa, Italy

**Keywords:** natural killer cells, dendritic cells, humans, cross-priming, Th1 cells

## Abstract

In recent years, the essential role of bi-directional cross-talk between natural killer (NK) and dendritic cells (DC) during immune responses has been clearly elucidated. In particular, this cross-talk results in the development of an efficient innate response, through DC-mediated NK cell activation, and a potent adaptive immune response, through NK-mediate DC editing and maturation. Recently, some novel human DC subsets have been identified: migratory DCs in afferent lymph and draining lymph nodes; CLEC9A^+^/BDCA3^+^ (CD141) DCs in interstitial dermis, liver, lung; *inflammatory* DCs in several inflammatory fluids. At the same time, it has been shown that also human NK cells are present in these compartments. Here, we will review the most recent findings on NK/DC cross-talk and we will discuss the necessity of acquiring more complete knowledge about these interactions in view of the new information available on both DC and NK cell subsets.

## Introduction

Natural killer (NK) cells were originally identified as lymphocytes that can spontaneously kill certain tumor target cells in the absence of previous stimulation *in vivo* or *in vitro* (1). NK cell activation results from the balance of signals produced by activating (2) and inhibitory (3) receptors. CD16 (FcRIIIa) is one of these activating NK cell receptors and binds human immunoglobulins, therefore mediating antibody-dependent cellular cytotoxicity (ADCC) of opsonized target cells. However, many other innate receptors acting upstream of the adaptive immunity have also been discovered. Among these, the first to be identified were natural cytotoxicity receptors (NCR) termed NKp46, NKp44, and NKp30 (2). NK cells also express additional activating receptors such as NKG2D and DNAM-1, which are partially shared with T lymphocytes, 2B4, NTBA, and NKp80 which promote NK cell triggering during the process of natural cytotoxicity (4). Activating NK cell signals are therefore mediated by several receptors and it is widely accepted that the ligands for NK cell activating receptors are mainly expressed on “stressed” cells, hence favoring killing of both tumor or infected cells (4). Nevertheless, an important exception to this rule is the ability of NK cells to kill normal autologous dendritic cells (DCs) (5, 6) as well as other immune cells such as macrophages and T lymphocytes (7–9).

On the other hand, human NK cells also express different inhibitory receptors recognizing human leukocyte antigen (HLA) class I molecules: killer immunoglobulin (Ig)-like receptors (KIRs) are specific for allelic determinants of HLA class I molecules, the Ig-like transcript (ILT)-2 receptor is characterized by a specificity for different HLA class I molecules, and CD94/NKG2A recognizes non-classical HLA class I molecules HLA-E (4). Therefore, cells that have lost HLA class I molecules such as tumor or virus-infected cells fail to deliver inhibitory signals to NK cells.

Peripheral blood NK cells in humans can be divided into two main subsets according to CD56 expression, namely CD56^dim^ and CD56^bright^, characterized by distinct functional and phenotypic properties. It has been established that a division of labor exists among these two subsets: CD56^dim^, expressing CD16, KIRs, and high levels of perforin, have enhanced killing activity, whereas CD56^bright^ cells, characterized by low levels of perforin and CD16, no KIRs and high expression of NKG2A, can secrete large amounts of cytokines (e.g., IFN-γ, GM-CSF, TNF) but not kill target cells. Nevertheless, with the appropriate stimulus, also CD56^dim^CD16^+^ NK cells are abundant cytokine producers (10, 11).

In the last few years, the functional links between NK cells and DCs have been widely investigated and different studies have demonstrated that reciprocal activations ensue upon NK/DC interactions. More recently, the anatomical sites where these interactions take place have started to be identified together with the related cell subsets involved.

Dendritic cells were identified for the first time in 1973 by Ralph Steinman as accessory cells in mice spleen. During the last two decades, it has been established that DCs are professional antigen presenting cells (APCs), uniquely skilled to attract and activate CD4^+^ and CD8^+^ T cells. Most of our knowledge on DCs comes from studies of blood and skin DCs. However, improvements of both flow cytometric and genomic approaches have recently allowed the identification of several distinct subsets of DCs. Despite their heterogeneity, there are some features common to all DC subsets, both in mice and humans. Immature DCs act like sentinels efficiently sampling antigenic material. Upon pathogen encounter, they undergo a complex maturation process that leads to professional antigen presentation, cytokine production, and T cell stimulatory capacities. During the maturation process, they upregulate distinct molecules on their surface such as major histocompatibility complex (MHC) class II, CD80, CD83, CD86, and CD40 essential for antigen presentation and interaction with T cells; at the same time, they migrate from the periphery to secondary lymphoid organs (SLO) where they can induce CD8^+^ and CD4^+^ T cell response (12).

Two main populations of DCs have been described in humans: BDCA2^+^ (CD303)/CD123^+^ plasmacytoid DCs (pDCs) and myeloid DCs (mDCs) (13). The latter includes several subsets identified in distinct tissues, thus resulting in a high level of heterogeneity; peripheral blood contains two main DC subsets: BDCA1^+^(CD1c) DCs and CLEC9A^+^/BDCA3^+^ (CD141) DCs (14, 15); as they are both also present in lymph nodes and tonsils, they have been described as blood-derived lymphoid organ-resident DCs (14–17).

Besides peripheral blood, also the skin includes distinct and well-characterized DC subsets: the epidermis contains Langerhans cells (LCs) and the dermis at least three subsets: dermal CD1a^+^ DCs, dermal CD14^+^ DCs, and CLEC9A^+^/BDCA3^+^ DCs (18–20). All these DC subsets can migrate through the lymph to draining lymph nodes (21). Finally, in several inflammatory conditions such as atopic dermatitis, psoriasis, rheumatoid arthritis, and tumor ascites, a different DC subtype, referred to as “inflammatory DC,” has lately been described (22). Transcriptomic analysis revealed that they likely derive from monocytes that differentiate at the site of inflammation. Interestingly, it has been recently suggested that, in inflamed tissues, CD56^bright^ NK cells may induce differentiation of monocytes into inflammatory DCs (23).

This broad heterogeneity corresponds to distinct specialized functions in terms of tissue distribution, cytokine release, antigen presentation, and regulation of T cell response. These different features of the distinct DC subsets will be reviewed here and discussed in the context of possible interactions of NK cells with different DC subsets.

## Distribution of Human NK Cell Subsets

In the last few years, it has become evident that NK cells are not exclusively found in peripheral blood and SLO but can populate different non-lymphoid tissues (24). In mice, where investigating NK cell localization is more straightforward than in humans, the presence of NK cells in many organs has been revealed (25) and distribution seems to be subset-specific, as different NK cell subsets showed organ-specific localizations. Lately, some light has also been shed regarding the distribution of human NK cells in solid tissues, showing that NK cells populate, and may re-circulate through most human peripheral tissues, and that organ-specific chemokine expression patterns can drive the homing of functionally distinct NK cell subsets to the various human body compartments, both at steady-state and pathology (26). In particular, CD56^bright^ NK cells selectively accumulate in several organs, including SLO, liver, visceral adipose tissues, and gastrointestinal tract. Moreover, in a large variety of human malignancies, CD56^bright^ NK cells represent the majority of NK cells infiltrating the tumor. Recently, we have reported that seroma, an accrual of fluid subsequent to surgical procedures such as axillary lymph node dissection, represents an accumulation of afferent lymph, drained from upstream tissues during the interval of time needed for lymphatic vessels to re-anastomose with the efferent ducts (27). Seroma accumulates without major contamination by either surgery-induced exudate or leaky blood-derived cells, thus confirming the lymph-associated origin of the cells contained in seroma fluids (21). Interestingly, only CD56^bright^/CD16^low/neg^/KIR^neg^ non-cytotoxic NK cells were detectable in afferent lymph from seroma fluids and appear therefore able, similarly to naïve T cells, to re-circulate via afferent lymph. NK cells are also present in human efferent lymph (28) suggesting that they can re-circulate from solid tissues to peripheral blood through lymphatic circulation and SLO.

The evidence that CD56^bright^ NK cells are, in most solid tissues, more abundant than in peripheral blood (which contains only around 2% of human body total lymphocytes) (29, 30), suggests they might probably outnumber CD56^dim^ NK cells in the human body. The functional role of such an abundant non-cytolytic, but cytokine-secreting, NK cell subset in solid organs remains to be fully clarified. Interestingly, it has been shown that human DCs primarily activate this NK cell subset (31) promoting IFN-γ release and proliferation.

## Distribution of Human DC Subsets

Thanks to improvements in both flow cytometric and genomic techniques, it is now clearer and clearer that human DCs represent a heterogeneous cell population and that each DC subset is often characterized by specific functional properties. BDCA1^+^ DCs have recently been described as the most potent human IL-12-producing APCs (32), suggesting a potential key role in promoting IFN-γ release by NK cells, and therefore Th1 polarization. CLEC9A^+^/BDCA3^+^ DCs, originally identified in peripheral blood and lymph nodes, have recently been detected also in other human organs such as skin, liver, lung, and intestine, where they show a more mature phenotype compared to CLEC9A^+^/BDCA3^+^ DCs observed in either blood or lymph nodes, indicating that they may represent a mature stage of differentiation (18). Moreover, CLEC9A^+^/BDCA3^+^ DCs are characterized by the peculiar ability to cross-present antigens from dead cells better than other DC subtypes (18) but they seem equally able to cross-present soluble antigens (33) when compared to other DCs. It can be hypothesized that different DC subsets need distinct TLR stimulation to efficiently cross-present exogenous antigens. Cross-presentation represents a key process for specific CTL response against most tumors and viruses that do not infect APCs. The antigen forms, as well as the activation signals received by DCs, are likely to be critical in determining the efficiency of cross-presentation. Moreover, it has been shown that CLEC9A^+^/BDCA3^+^ DCs have the dual capacity to produce both IL-12 and type I IFN (34), thereby enabling both NK cell activation and Th1 polarization, which could be significant for a protective immune response against viral infections. In particular, type I IFN can enhance NK cell cytotoxicity while IL-12 promotes IFN-γ secretion and Th1 polarization.

Dendritic cells in human skin also show distinct patterns of functional capabilities: dermal CD1a^+^ DCs have been described as immature cells capable of inducing T cell response only upon stimulation, while in steady-state they might be tolerogenic (35). On the other hand, dermal CD14^+^ DCs seem to play a critical role in the regulation of humoral immunity and LC in the induction of CTL response (20). LCs also secrete IL-15 and are therefore potentially able to activate both CD8^+^ T cells and NK cells (36).

In general, immature (non-activated) DCs act as sentinels in peripheral tissues; upon activation through danger signals they initiate the maturation process that allows them to migrate to lymph nodes via afferent lymph. In many experimental animal models, DCs have been shown to be able to continuously migrate from intestine or from skin to SLO (12, 37, 38). Most recently, DCs in human afferent lymph have also been characterized (21). Besides dermal CD1a^+^ DCs, dermal CD14^+^ DCs and LC, afferent lymph also includes CLEC9A^+^/BDCA3^+^ DCs and CD1a^+^ CD14^+^ DCs, the latter likely representing an immature stage of differentiation from CD14^+^ DCs to CD1a^+^ DCs.

## NK Cell Cross-Talk with Dendritic Cells

The cooperative interaction between DCs and NK cells plays a key role in triggering immune response against pathogens. This dialog results in a bi-directional activation and has effects also on the subsequent adaptive immune response, influencing the development of Th1 cells and CTLs, both essential for an effective anti-tumor and anti-viral immune response.

### DCs induce NK cell activation

**Figure 1:**
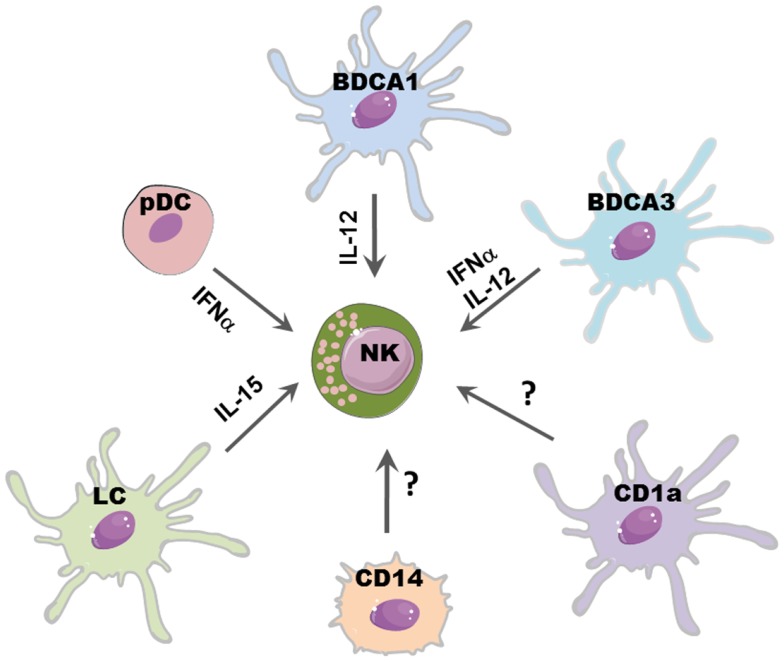
**DCs subsets may differently affect NK cell function**. Distinct DC subsets reflect different capability to promote NK cell function: IL-15 released by LC may promote NK cell proliferation; IL-12, mainly produced by CLEC9A^+^/BDCA3^+^ DCs and BDCA1^+^ DCs, induces IFN-γ release and subsequent Th1 polarization of T cells; NK cytolytic activity may be boosted by IFN-α secreted by pDCs and CLEC9A^+^/BDCA3^+^ DCs.

Dendritic cells promote the release of cytokines by NK cells (mainly TNF and IFN-γ) and enhance NK cell proliferation and cytolytic activity. DC-mediated NK cell activation occurs mainly through the release of soluble factors (Figure 1) although cell-to-cell contacts play a relevant role during NK/DC interaction, as better specified below.

It has been shown, in different mice models, that NK cell pre-activation by DCs is required for an efficient immune response against viral infections (39–41) and tumors (42). A large variety of microbial stimulation and signaling via TLRs can induce DC maturation and secretion of several cytokines which can in turn activate NK cells. IL-12, mainly secreted by mDCs (in particular from BDCA1^+^ DCs), efficiently stimulate IFN-γ secretion by NK cells. IL-18 can potentiate the effect of IL-12 by inducing the expression of IL-12R on NK cells. Moreover, IL-18 synergizes with IL-12 for enhancing NK cell cytolytic activity (43).

Also pDCs might activate NK cells, most likely via the release of type I IFN, which has been shown sufficient to boost NK cell cytotoxicity (44). Indeed, a protective NK cell response during infection with the murine cytomegalovirus (MCMV) was found to be type I IFN-dependent (45). Of note, the recently described CLEC9A^+^/BDCA3^+^ DC subset can also release high amounts of INF-α, suggesting that, upon viral infection, they may play a key role in promoting NK cell cytotoxicity in peripheral tissues, such as skin, liver, lung, and intestine (34).

Another relevant cytokine for NK cell development and functions is IL-15, which is also produced by DCs. This cytokine can be presented by DCs via its binding to IL15R alpha or as transmembrane protein; it can stimulate NK cell proliferation, survival, and priming of protective NK cell response (44). In particular, it has been shown that LC can support NK survival via IL-15 (46). Besides the membrane-bound form of IL-15, it has been established that also other contact-dependent mechanisms are involved in NK–DC cross-talk. In general, the formation of stimulatory synapses between DCs and NK cells plays a critical role during NK cell activation induced by DC-derived cytokines, including IL-12 (47). Also, the interaction of CXC3CL1 expressed on DCs with CX3CR1 on NK cells results in IFN-γ release by NK cells (48) and it has been shown that influenza virus-infected DCs can support IFN-γ production by triggering the activating receptors NKp46 and NKG2D (49).

Most of the studies on NK/DC interactions in humans are based on DCs derived from monocytes, which are generated after several days of culture with different cytokines. On the other hand, the interactions between *ex vivo* isolated human DCs and NK cells have been poorly investigated so far and, despite the clear heterogeneity of human DC subsets, only peripheral blood DCs have, to some extent, been investigated (50–52). In these studies, it has been shown that both plasmacytoid and myeloid peripheral blood DCs are capable of activating NK cells, enhancing their cytolytic activity and inducing IFN-γ release in response to influenza virus or dsRNA.

Among human NK cell subsets, CD56^bright^ NK cells were found to be particularly responsive to activation by DCs (31, 53). Interestingly, they are enriched in SLO and in most solid tissues (26). Their presence in afferent lymph also suggests that they may re-circulate from peripheral solid tissues to SLO; thus, it is conceivable that, *in vivo*, NK–DC cross-talk may occur either in peripheral tissues or in lymph nodes, where, in both cases, NK cells can encounter distinct myeloid DC subsets. Recent reports indicate that DC heterogeneity may also correspond to the induction of different functions in NK cells: BDCA1^+^ DCs may be important for IL-12 secretion in SLO, favoring IFN-γ secretion and consequent Th1 polarization of T cells; CLEC9^+^BDCA3^+^ DCs may be relevant in peripheral tissue where, upon virus infection, they can induce NK cytolytic activity by releasing IFN-α.

**Figure 2:**
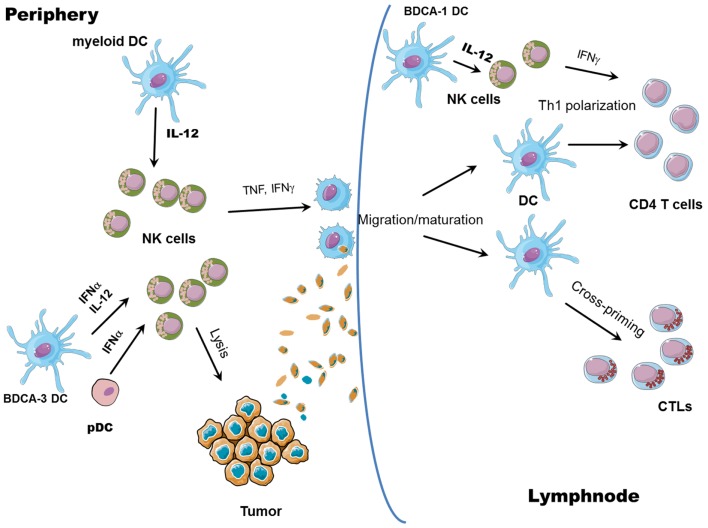
**NK/DC cross-talk**. The interaction between NK cells and DCs results in reciprocal activation: mature DCs release cytokines able to promote NK cell activation (all myeloid mature DCs can produce IL-12 whereas pDCs and CLEC9A^+^ BDCA3^+^ DCs can release large amounts of IFN-α); in turn, IFN-γ released by activated NK cells promotes Th1 polarization and, together with TNF, DC maturation, and migration to draining lymph nodes. Activated NK cells can also lyse tumor cells, leading to the generation of tumor antigenic material; tumor debris are then engulfed by DCs and tumor antigens are processed and presented by mature DCs to both CD8^+^ (cross-priming) and CD4^+^ T cells.

In conclusion, the activation of NK cells ensuing upon interaction with DCs has important consequences not only for the lysis of tumor or virus-infected cells, but it can also boost ongoing adaptive responses by the release of IFN-γ, which promotes type 1 polarization of T cells (Figure 2). Moreover, once activated, NK cells can edit DCs, by eliminating the more immature, allegedly tolerogenic DCs, as further discussed below. At the same time, NK cells can also shape adaptive immune responses by causing DC activation.

### NK cells induce activation and editing of DCs

Activation of NK cells can occur via triggering of activating receptors by target cells or by stimulation of soluble factors released by accessory cells. Following activation, NK cells release large amounts of TNF and IFN-γ, which are known to affect DC maturation. TNF enhances the expression of costimulatory molecules on DCs and, synergizing with IFN-γ, contributes to DC production of IL-12 (54, 55). Moreover, exposure of NK cells to innate cytokines such as IL-12 and IL-18 (both released by mDCs) can promote Th1 polarization [Figure 2 and Ref. (56)]. INF-γ can also induce the expression of a membrane-bound form of IL-15 on DCs, thus sustaining both T and NK cell survival and activation (57).

Besides soluble factors, it has been shown that engagement of the NK activating receptor NKp30 can mediate DC maturation (58). Thus, recognition of target cells by NK cells can induce an additional mechanism of DC maturation, which might be particularly relevant in tumor immunity, where the absence of danger signals precludes DC maturation via the engagement of pattern recognition receptors (59, 60).

During initiation of an anti-viral or anti-tumor immune response, the microenvironment is influenced by a peculiar cytokine milieu, which includes cytokines released following NK cell activation (61, 62). It must be noted that NK cell triggering often occurs upstream of T cell activation, providing both a first line of defense and an early production of cytokine, critical for the subsequent development of the adaptive immune response. Although it is generally accepted that CTL response needs helper signals provided by CD4^+^ T cells, interactions occurring between DCs and NK cells can bypass these helper signals by leading to the production of IFN-γ, which, in turn, can stimulate IL-12 production by DCs, thus eventually leading to a protective CTL response (57, 63, 64).

While the helper role of NK cells in inducing DC-mediated generation of Th1 polarized T cells and CTLs has been well documented, an issue not exhaustively elucidated so far is the ability of NK cells to promote DC cross-priming. Nevertheless, some reports suggest a role for NK cells in promoting antigen cross-presentation by DCs. It has been shown that DCs can take up dying cells killed by NK cells and present them on MHC class I molecules (65, 66). Obviously, NK cell ability to lyse virally infected or tumor cells could help uptake and cross-presentation of antigens by DCs but whether NK cells also play a direct role in favoring DC cross-presentation is still not clear. In a human *in vitro* system, it has been demonstrated that cross-presentation of antigens to CD8^+^ T cells by DCs requires NK cells: capture of tumor cells and maturation status of DCs are not sufficient to induce cross-priming of T cells without further NK-mediated activation and IL-18 release (67). Moreover, the capability of mono-derived DCs, generated in the presence of IFN-α (IFN-DCs), to prime CD8^+^ T cells against human tumor antigens is dependent on NK cells; NK cell removal indeed leads to generation of IFN-DCs with no priming activity of tumor Ag-specific T cells (68). *In vivo*, in a mice model of melanoma, tumor regression resulted from an immune cascade initiated by activated pDCs and involving NK cells, mDCs, and T cells. It was shown that CpG-activated pDCs can recruit NK cells at the tumor sites via chemokine production (CCL3, CCL4, and CCL5), and enhance their cytolytic activity through IFN-α release. Activated NK cells, in turn, can kill tumor cells, induce mDC maturation, and migration to draining lymph nodes, where mDCs can cross-present tumor antigens to CD8 T cells (69). Again, in this study, cross-priming of CD8^+^ T cells is exclusively NK cell-dependent, as NK cell depletion results in complete abrogation of CD8^+^ T cell priming. Therefore, it is likely that NK cells can favor cross-presentation by DCs, although the specific abilities of different DC subsets, as well as the mechanisms involved are still to be clearly identified. It is conceivable that NK cell killing of tumor cells could provide antigens subsequently taken up, processed, and cross-presented by DCs; at the same time, activation of NK cells is associated to the secretion of cytokines, such as TNF or IFN-γ, potentially able to help cross-priming of specific CTLs (Figure 2).

Thus, NK cells, upon interaction with DCs, can induce the activation of specific functions on DCs. Nevertheless, the capability of NK cells to induce DC activation is not the only mechanism by which NK cells may influence DC functions. Once activated by DCs, NK cells acquire the capability of killing immature, but not mature, mDCs (5). It has been proposed that more mature, activated DCs, by upregulating their surface expression of MHC class I molecules, would be protected from NK cell lysis. Conversely, immature DCs, expressing lower levels of MHC class I molecules, are more susceptible to NK cell killing. DCs that fail to express sufficient amounts of MHC class I molecules would induce inappropriate, low affinity T cell priming resulting either in Th2 response or in the induction of tolerance (70, 71). For these reasons, it was hypothesized that NK-mediated DC killing might represent a mechanism of DC selection for the control of downstream adaptive immune response (DC editing) (70). While *in vivo* evidence for DC activation by NK cells has extensively been provided, the direct demonstration that DC editing by NK cells also occurs *in vivo*, as well as its putative role in promoting an efficient immune response, has only recently been proven (72). In an experimental model of cancer cell vaccination, NK cells were necessary for removing less immunogenic DCs by a perforin-dependent mechanism, leading to an improved capability of residual DCs to induce anti-tumor CTL response and mice survival.

## Concluding Remarks

Studies performed in the last few years have clearly shown that, during immune response, different leukocytes act by not only displaying their own protective functions, but also interacting with each other to optimize the response against microorganisms and cancer cells. Recent identification of different DC subsets in the human system is leading to new insights in the field of innate cell interactions, particularly for the cross-talk occurring between these DC subsets and NK cells. As DC subsets show a specific distribution in human tissues, their interactions with NK cells should now be better dissected. Noteworthy, some light has also been recently shed regarding the distribution and trafficking of NK cells in the human body, thus allowing a more complete depiction of where these two cell types could physically interact. Interestingly, DC subsets are now emerging as cells endowed with peculiar functions, either in terms of specific cytokine secretion or of signals provided to other neighboring cells through distinctive surface molecules during cell-to-cell contacts. Therefore, NK/DC interactions should no longer be considered as the cross-talk between two homogeneous populations of innate cells but rather as a more complex network of cell subset cooperation acting in discrete regions of the body to fulfill complementary tasks.

## Conflict of Interest Statement

The Review Editor Mariella Della Chiesa declares that, despite being affiliated to the same institution as author Barbara Morandi, the review process was handled objectively and no conflict of interest exists. The authors declare that the research was conducted in the absence of any commercial or financial relationships that could be construed as a potential conflict of interest.
